# Interprofessional diabetes and oral health management: what do primary healthcare professionals think?

**DOI:** 10.12688/f1000research.52297.1

**Published:** 2021-05-04

**Authors:** Phyllis Lau, Anthony Tran, Matthew Chen, Evelyn Boyce, Rachel Martin, Hanny Calache

**Affiliations:** 1 Department of General Practice, The University of Melbbourne, Melbourne, Victoria, 3010, Australia; 2 North Richmond Community Health, Richmond, VIC, 3121, Australia; 3 Rural Health School, La Trobe University, Flora Hill, VIC, 3550, Australia; 4 Institute of Health Transformation, Deakin University, Burwood, VIC, 3125, Australia

**Keywords:** interprofessional care, diabetes mellitus, oral health, periodontitis, primary healthcare, general practice, primary dental

## Abstract

**Background: **Diabetes and periodontitis have a bi-directional relationship. And yet, collaborations between primary healthcare practitioners in diabetes and oral health care are minimal. This study explored the views of general practice and oral health professionals on the link between diabetes and periodontitis, and interprofessional diabetes and oral health management.

**Methods:** A sequential mixed-methods exploratory research design was used. General practice and oral health professionals were recruited from four community health centres in Melbourne. Quantitative surveys explored participants’ experiences, attitudes and knowledge of diabetes and oral health management and interprofessional collaboration; qualitative follow-up interviews explored survey responses with selected participants.

**Results: **58 participants completed the online surveys; 22 then participated in semi-structured interviews. Participants generally had strong intentions to collaborate interprofessionally in diabetes and oral health management. Most general practice and oral health professional participants were willing to perform simple screening for periodontitis or diabetes respectively. Themes from the interviews were grouped under three domains: ‘a *ttitude towards diabetes and oral health management’, ‘subjective norms’ *and *‘perceived behavioural control’; *and an overarching domain to describe participants’ ‘current practice’. Existing siloed primary healthcare practices and lack of formal referral pathways contribute to poor interprofessional collaboration. Most participants were unsure of each other’s responsibilities and roles. Their lack of training in the relationship between general and oral health, compounded by systemic barriers including time constraint, high dental costs, long public dental waiting list and unintegrated health information systems, also impeded interprofessional care.

**Conclusions:** The diabetes and oral health link is not properly recognised or managed collaboratively by relevant primary healthcare professionals in Australia. There is, nonetheless, strong intentions to engage in interprofessional diabetes and oral health care to contribute to improved patient outcomes. Primary healthcare professionals need dedicated and accredited interprofessional training and competencies, formal referral systems and sustainable health policies to facilitate collaboration.

## Introduction

Current evidence shows a bidirectional link between diabetes and chronic periodontal disease (periodontitis). Diabetes is associated with increased risk of an inflammatory response to periodontal micro-biota. Severe periodontitis is three- to four-times more prevalent in people with diabetes. Periodontitis on the other hand seems to affect blood glucose levels in patients with diabetes. Severity of periodontitis may be associated with increased diabetes episodes requiring hospitalisation ^1– 3^. People with both diabetes and periodontitis have increased risk of premature tooth loss, poorer diet, poorer diabetes control and more cardiovascular complications. In the 1990s, chronic periodontitis was added as the sixth complication of diabetes mellitus ^2^.

Both Australian and international guidelines recommend that diabetes care providers should undertake oral health reviews and provide dental referrals if required. The Royal Australian College of General Practitioners (RACGP) recommends to assess the oral health of patients with diabetes ^4^. The International Diabetes Federation (IDF) recommends the strengthening of interdisciplinary collaboration to improve general patient outcomes and as a primary means to prevent periodontitis for patients with diabetes ^5^. Despite these recommendations, the potential for screening patients with diabetes for early management of gum problems is often overlooked in primary care. There are siloes in practice and a lack of collaboration between general practice and oral health professionals ^6– 8^. Consistent with current guidelines, general practitioners (GPs) usually prescribe short-term pain relief and/or antibiotics for teeth and gum issues and may advise patients to see a dentist. This is usually the extent of their involvement with oral health management. Similarly, diabetes screening is rarely performed by OHPs despite research showing significant proportions of dental patients have undiagnosed diabetes or pre-diabetes risks ^3, 9, 10^.

Much of the current literature nonetheless focuses on assessment of diabetes screening in dental settings or the evolution of the dental profession ^3, 9, 11^. Some have also explored the views of healthcare professionals on integration of diabetes and periodontitis management including some recent work conducted at the Centre for Oral Health Outcomes, Research Translation and Evaluation at Western Sydney University in New South Wales ^12– 17^. However, limited research on interprofessional diabetes and oral health care has been conducted in Victoria or focused on public community health service (CHS) setting.

This study aimed to explore the knowledge, practice and attitude of Victorian general practice professionals (GPPs) [including GPs, primary health care nurses (PCNs), diabetes educators (DEs)] and oral health professionals (OHPs) [including dentists (Ds), dental hygienists (DHs), oral health therapists (OHTs), dental therapists (DTs) and dental assistants (DAs)] in managing diabetes and periodontitis and their views on interprofessional care in CHS setting.

## Methods

### Ethics approval

This research was approved by human research ethics committees at University of Melbourne (ID 1750835), Deakin University (ID 2018-190) and La Trobe University (ID 1750835). 

Written informed consent from the participants for the publication of findings from this research was obtained. In accordance with the requirements of the ethics committee and the approved research protocol, details that would potentially identify participants due to the small sample size have been removed or replaced with codes in this publication.

### Study design

This is a sequential mixed-methods exploratory study. Quantitative online surveys and qualitative semi-structured interviews were conducted with healthcare professionals. Quantitative analysis reported descriptive statistics only. Qualitative analysis used a mixed inductive and deductive approach to explore the experiences of healthcare professionals and reporting was guided by the consolidated criteria for reporting qualitative research checklist (COREQ) ^18^.

### Advisory group

An advisory group guided the implementation of the study. It consisted of representatives from consumers, practitioners (GP, DE, general dentist, periodontist, oral health therapist) and managers of the CHSs involved.

### Research team

PL is an academic primary care researcher; EB is a diabetes nurse educator; HC is an academic dental public health researcher; MC and AT were honours research students and RM was a public general dentist at the time of the project. The team has an interest in promoting interprofessional primary healthcare.

### Setting and participant recruitment

GPPs and OHPs were recruited purposively from four CHSs in Victoria. The project was presented by the research team to eligible staff at two CHSs; email invitations with a short introduction video were sent to eligible staff *via* their management teams at the other two. Staff were assured that participation was voluntary. Project description and an anonymous survey link were given to all participants.

On completion of the survey, participants’ contact details were sought if they opted to participate in follow-up interviews. A matrix (gender, age, professional role) was used to select, for the interviews, a broad representation of those who provided contact details to ensure maximum variation.

### Quantitative surveys

Two online surveys (one for GPPs; one for OHPs) were conducted *via* the REDCap electronic data capture tool hosted at The University of Melbourne platform ^19^. The questions ( Table 1 and Table 2) were developed based on a review of the literature and guided by our advisory group. They were piloted with GP registrars, academic nurses and dentists in the research team’s network before the surveys were rolled out.

**Table 1.** Survey questions for GPPs.

**Demographics** 1. Your gender: male, female 2. Your age: under 30, 31–40, 41–50, 51–60, over 60 3. Please state your profession: general practitioner (GP) or primary care nurse (PN), diabetes educator, other (If other, please specify) 4. Your current primary workplace where you practice as a primary healthcare professional (PHP): public, private, other (If other, please specify) 5. Your current primary workplace is located in a community that is: rural, metropolitan 6. The approximate number of hours you currently practise as a PHP in a week: 0–10, 11–20, 21–30, 31–40, more than 40. 7. The number of years you have been practising as a PHP: under 5, 6–10, 11–15, 16–20, 21–25, 25–30, over 30
**Education and Training** 8. Your medical or nursing training was received in: Australia, Overseas (If overseas, please specify where) 9. In your view, did you find the oral health and education components of your medical or nursing course thorough enough?: Yes, No, Not sure 10. How would you typically find information on oral health?: Internet, Professional magazines, journals, your colleagues, continuing professional education, other (If other, please indicate where) 11. Would you welcome the opportunity for continuing education and training in oral health?: Yes, No, Not sure
**Current Practice and Attitude** 12. How confident are you in identifying the signs and symptoms associated with: • gingivitis? Very confident, confident, not confident, not confident at all • periodontitis? Very confident, confident, not confident, not confident at all • oral disease in general? Very confident, confident, not confident, not confident at all 13. On average, how many patients would you see in a week who either present with or complain of gum disease or other oral health conditions?: 0, 1–10, 11–20, 21–30, 31–40, more than 40 14. On average, how many patients would you see in a week who present with type 2 diabetes or risks of diabetes?: 0, 1–10, 11–20, 21–30, 31–40, more than 40 15. On average, how many patients with type 2 diabetes or risks of diabetes would you see in a week who also have issues with their gum or other oral health issues?: 0, 1–10, 11–20, 21–30, 31–40, more than 40, don’t know 16. How confident do you feel about managing a patient with diabetes or risks of diabetes and oral health issues (including periodontal disease) in your practice? Very confident/Confident/Not confident/Not confident at all 17. Are you aware of the two-way link between oral health and type 2 diabetes?: Yes, No 18. How comfortable are you to broach the subject of oral health with a patient who has come to see you about their type 2 diabetes or risks of diabetes?: Very comfortable, comfortable, not comfortable, not comfortable at all 19. Are you aware of the following oral behaviors and/or conditions that could impact on the management of Type 2 diabetes?: i. poor oral hygiene: Yes, No, Not sure ii. eating certain foods: Yes, No, Not sure iii. drinking alcohol: Yes, No, Not sure iv. smoking: Yes, No, Not sure v. gingivitis: Yes, No, Not sure vi. periodontitis: Yes, No, Not sure vii. tooth decay: Yes, No, Not sure viii. food trapping: Yes, No, Not sure ix. dental infection: Yes, No, Not sure 20. How often do you conduct oral investigations on patients with type 2 diabetes or risks of diabetes and suspected oral health conditions?: Always, Often, Occasionally, Rarely, Never. 21. How often do you refer patients with type 2 diabetes or risks of diabetes to an oral health professional for the management of their oral health conditions?: Always, Often, Occasionally, Rarely, Never 22. In your view, is it within your role as a PHP to undertake oral health screening for your patients with type 2 diabetes or risks of diabetes?: Yes, No, Not sure 23. It is envisaged that an oral health screening tool for GPs and PNs is likely to involve a visual non-invasive inspection with a torch and approximately 5 screening questions. How comfortable would you be to use such a tool?: Very comfortable, comfortable, not comfortable, not comfortable at all 24. How much do you agree or disagree with the following statement? It would be feasible in my practice to conduct simple oral health screening (with training) for my patients with type 2 diabetes or risks of diabetes.: Strongly Agree, Agree, Neutral, Disagree, Strongly Disagree. 25. How much do you agree or disagree with the following statement? The clinical staff in my practice would welcome the introduction of a simple oral health screening tool for patients with type 2 diabetes or risks of diabetes.: Strongly Agree, Agree, Neutral, Disagree, Strongly Disagree. 26. In your view, would better collaboration between general practice and dental staff benefit patients with type 2 diabetes or risks of diabetes and oral health problems?: Strongly Agree, Agree, Neutral, Disagree, Strongly Disagree 27. Do you think there is a role for Oral Health Practitioners to screen for diabetes in their dental patients?: Yes, No, Not sure 28. Would you be willing to undertake educational training to assist you in providing oral health advice for your patients type 2 diabetes or risks of diabetes? Yes, No

**Table 2.** Survey questions for OHPs.

**Demographics** 1. Your gender: male, female 2. Your age: under 30, 31–40, 41–50, 51–60, over 60 3. Please state your profession: dentist, dental therapist, dental hygienist, oral health therapist, dental assistant Cert IV in Health promotion, other (If other, please specify) 4. Your current primary workplace where you practise as an oral health professional (OHP): public, private, other (If other, please specify) 5. Your current primary workplace is located in a community that is: rural, metropolitan 6. The approximate number of hours you currently practise as a OHP in a week: 0–10, 11–20, 21–30, 31–40, more than 40. 7. The number of years you have been practising as an OHP: under 5, 6–10, 11–15, 16–20, 21–25, 25–30, over 30
**Education and Training** 8. Your oral health training was received in: Australia, Overseas (If overseas, please specify where) 9. In your view, did you receive appropriate education and training in your oral health course regarding the connection between oral health and diabetes?: Yes, No, Not sure 10. How would you typically find information on medical conditions that impact on your treatment of oral health? Internet, Professional magazines, journals, your colleagues, continuing professional education, other (If other, please indicate where) 11. Would you welcome the opportunity for continuing education and training in the links between oral health and diabetes?: Yes, No, Not sure
**Current Practice and Attitude** 12. How confident are you in identifying the risk factors associated with type 2 diabetes? Very confident/Confident/Not confident/Not confident at all 13. Are you aware of the following risk factors for type 2 diabetes?: i. poor oral hygiene: Yes, No, Not sure ii. eating certain foods: Yes, No, Not sure iii. drinking alcohol: Yes, No, Not sure iv. smoking: Yes, No, Not sure v. gingivitis: Yes, No, Not sure vi. periodontitis: Yes, No, Not sure vii. dental infection: Yes, No, Not sure 14. On average, how many patients would you see in a week who present with periodontal disease? 0, 1–10, 11–20, 21–30, 31–40, More than 40 15. On average, how many patients with periodontal disease would you see in a week who also have diabetes or risk factors associated with type 2 diabetes?: 0, 1–10, 11–20, 21–30, 31–40, More than 40, Don’t know 16. How confident do you feel about managing a dental patient with risk factors for diabetes (including periodontal disease) in your practice? Very confident/Confident/Not confident/Not confident at all 17. Are you aware of the two-way link between oral health and type 2 diabetes? Yes, No 18. How comfortable are you to broach the subject of diabetes with a patient with periodontal disease? Very comfortable/Comfortable/Not comfortable/Not comfortable at all 19. How often do you consult with GPs on patients with periodontal conditions and suspected diabetes?: Always, Often, Occasionally, Rarely, Never. 20. How often do you refer patients who present with periodontal disease and suspected diabetes to a GP?: Always, Often, Occasionally, Rarely, Never 21. In your view, is it within your role as a OHP to undertake diabetes screening for your patients with periodontal conditions? Yes, No, Not sure 22. Are you aware of the AUSDRISK tool for assessing the diabetes risk status of patients? Yes, No, Not sure 23. How much do you agree or disagree with the following statement? It would be feasible in my practice to use the AUSDRISK screening tool (10 questions) for my patients with periodontal disease. Strongly agree/Agree/Not sure/Disagree/Strongly disagree 24. How much do you agree or disagree with the following statement? The clinical staff in my practice would welcome the introduction of the AUSDRISK screening tool (10 questions) for my patients with periodontal disease. Strongly agree/Agree/Not sure/Disagree/Strongly disagree 25. In your view, would better collaboration between general practice and dental staff benefit patients with risk factors for Type 2 diabetes (e.g. periodontal disease)? Yes, No, Not sure 26. Do you think there is a role for general practice professionals to screen for risk of periodontal disease in their patients with diabetes or risks of diabetes? Yes, No, Not sure 27. Would you be willing to undertake educational training to assist you in providing advice for your dental patients who also have type 2 diabetes or risk factors for type 2 diabetes (e.g. periodontal disease)? Yes, No

Likert scales gauged participants’ agreement with statements relating to confidence, current practice and interprofessional collaboration in oral health or diabetes management and perceived feasibility of screening for periodontitis or diabetes within routine practice. Data were analyzed in Microsoft Excel (2017) (RRID:SCR_016137) to produce descriptive statistics. Google Sheets ( **RRID:SCR_017679**) is a free alternative.

### Qualitative interviews

Participants were asked to opt into interviews to explain their survey responses, identify barriers to diabetes and oral management, and suggest ways to improve interprofessional diabetes and oral health management ( Table 3 and Table 4). Author AT interviewed GPPs whilst author MC interviewed OHPs either by phone or in-person at the participants’ practice. Both were trained by author PL in interview techniques and did practice interviews with authors PL and HC. Questions were pilot tested with students in the Department of General Practice Honours student cohort prior to conducting the interviews.

**Table 3.** Interview questions for GPPs.

1. In the survey, you indicated that you are **very comfortable/comfortable/not comfortable/not comfortable at all** about broaching the subject of oral health with a patient with type 2 diabetes or risks of diabetes. Please tell me more about what you meant. • Prompt: What are some barriers you have experienced when broaching the subject of oral health with a patient with type 2 diabetes or risks of diabetes? 2. In the survey, you indicated that you **always/often/occasionally/rarely/never** conduct oral investigations on patients with type 2 diabetes or risks of diabetes and suspected oral health conditions. Please explain more. 3. How confident do you feel about managing a patient with type 2 diabetes or risks of diabetes and oral health problems in your practice? Please explain more. • Prompt: What are some barriers you have experienced when managing a patient with diabetes or risks of diabetes and oral health problems? 4. You have indicated that with patients with diabetes or risks of diabetes who have oral health problems, you **always/often/** **sometimes/rarely/never** refer them to an OHP. Please tell me more about why this is so. 5. In the survey, you strongly **agree/agree/neutral/disagree/strongly disagree** that better collaboration between GPs, PNs & OHPs would benefit patients with type 2 diabetes or risks of diabetes and oral health problems. Would you please explain? • Prompt: What are your thoughts on possible ways to improve the collaboration between GPs/PNs and OHPs regarding the management of diabetes and periodontal disease? 6. What other suggestions do you have that would enable a GP to better manage patients with diabetes or risks of diabetes and oral health problems? 7. In the survey, an oral health screening tool was mentioned, which involves a visual non-invasive inspection with a torch and a series of approximately 5 screening questions. What concerns would you have regarding the feasibility of implementing this screening tool to patients with type 2 diabetes or risks of diabetes in your practice? 8. What are your concerns regarding the acceptability of your staff in implementing this screening tool to patients with diabetes or risks of diabetes in your practice? 9. How much time would you be willing to devote to training and education related to the impact of oral health on the management of diabetes and with regards to the implementation of the proposed oral health screening tool? 10. What do you think is the role for Oral Health Practitioners in assessing the type 2 diabetes risk status of their dental patients and then referring for management by a GP? Why do you say that?

**Table 4.** Interview questions for OHPs.

1. In the survey, you indicated that you are **very comfortable/comfortable/not comfortable/not comfortable at all** about broaching the subject of the risk of Type 2 diabetes with a patient who has periodontal disease. Please tell me more about what you meant. • Prompt: What are some barriers you have experienced when broaching the subject of the risk of Type 2 diabetes with a patient who has periodontal disease? 2. In the survey, you indicated that you are **very confident/confident/not confident/not confident at all** at managing a dental patient with risk factors for diabetes, such as periodontal disease. Please explain more. • Prompts: What are some barriers you have experienced when managing a dental patient with risk factors for diabetes (including periodontitis) in your practice? 3. In the survey, you indicated that with patients who have periodontal disease and suspected diabetes, you **always/often/** **sometimes/rarely/never** refer them to a GP. Please tell me more about why this is so. 4. In the survey, you considered it **within/not within** your role as an OHP to undertake diabetes screening for patients with periodontal conditions. Please explain more. 5. In the survey, you **strongly agree/agree/neutral/disagree/strongly disagree** that better collaboration between GPs, PNs and OHPs would benefit patients with risk factors for diabetes? Would you please explain? • Prompt: What are your thoughts on possible ways to improve the collaboration between GPs/PNs and OHPs regarding the management of diabetes and periodontal disease? 6. What other suggestions do you have that would enable an OHP to better manage patients with risk factors for diabetes? 7. In the survey, you indicated **your familiarity/unfamiliarity** with the AUSDRISK Type 2 diabetes screening tool. What concerns would you have regarding the feasibility of implementing this screening tool to patients with periodontitis in your practice? 8. What are your concerns regarding the acceptability of your staff in implementing the AUSDRISK Tool to patients with periodontal disease in your practice? 9. How much time would you be willing to devote to training and education with regards to the impact of Type 2 diabetes on the management of patients with periodontal disease, as well as the implementation of the AUSDRISK tool in your practice? 10. What do you think is the role for general practice staff in assessing the oral health of their patients with type 2 diabetes and then referring for management by an OHP? Why do you say that?

Interviews were audio-recorded and transcribed, and field notes were taken. Transcripts were offered to participants for review before being imported into QSR International's NVivo 12 qualitative analysis software (RRID:SCR_014802) ^20^. RQDA package for R ( **RRID:SCR_001905**) is an open-source alternative. AT coded all GPP interviews, MC coded all OHP interviews while the rest of the team (PL, EB, RM and HC) coded up to six interviews each, ensuring every transcript was coded by at least two researchers. Transcripts were first inductively coded separately and then collectively by the research team. Following several iterative meetings to reach consensus in coding and categorising differences, the research teams decides that the Theory of Planned Behavior model (TPB) which outlines three domains affecting intention to perform a behavior: attitude towards the behavior (or beliefs which influence an individual to perform a behavior), subjective norms (or perceived external pressures as influenced by judgement of others) and perceived behavioral control (or ease or difficulty in performing the behavior as determined by external factors) is congruent with patterns emerging ^21^. Deductive analysis using a framework analysis approach then followed using the TPB to identify patterns and elicit themes ^22^. The team continued to meet to discuss the themes elicited until agreement was reached.

## Results

### Survey participant demographics

A total of 58 participants completed the survey between April and July 2018: 20 from general practice (eight GPs, nine PCNs and three DEs) and 38 from dental practice (18 Ds, four DHs, six OHTs, five DTs and five DAs). ( Table 5)

**Table 5.** Survey participants’ demographics.

Variables	General practice professionals (n=20) No. (%)	Oral health professionals (n=38) No. (%)	Total (n=58) No. (%)
**Gender**			
Male	6 (30%)	4 (11%)	10 (17%)
Female	14 (70%)	32 (84%)	46 (79%)
Did not indicate	0	2 (5%)	2 (3%)
**Age in years**			
<30	0	9 (24%)	9 (16%)
31–40	5 (25%)	15 (39%)	20 (34%)
41–50	1 (5%)	8 (21%)	9 (16%)
51–60	10 (50%)	4 (11%)	14 (24%)
>60	4 (20%)	2 (5%)	6 (10%)
**Hours worked per week (h/w)**			
0 – 10	1 (5%)	8 (21%)	9 (16%)
11 – 20	3 (15%)	1 (3%)	4 (7%)
21 – 30	8 (40%)	9 (24%)	17 (29%)
31 – 40	8 (40%)	16 (42%)	24 (41%)
>40	0	4 (11%)	4 (7%)
**Years in experience**			
0 – 10	4 (20%)	20 (53%)	24 (41%)
11 – 20	7 (35%)	8 (21%)	15 (26%)
21 – 30	8 (40%)	8 (21%)	16 (28%)
>30	1 (5%)	2 (5%)	3 (5%)
**Professional training received in**			
Australia	15 (75%)	28 (74%)	43 (74%)
Overseas	5 (25%)	10 (26%)	15 (26%)
**Number of patients seen per week with** **oral health issues**			
0	6 (30%)	N/A	-
1–10	13 (65%)		-
11–20	1 (5%)		-
21–30	0		-
31–40	0		-
>40	0		-
Don’t know	0		-
**Number of patients seen per week with** ** type 2 diabetes**			
0	1 (5%)	N/A	-
1–10	8 (40%)		-
11–20	8 (40%)		-
21–30	3 (15%)		-
31–40	0		-
>40	0		-
Don’t know	0		-
**Number of patients seen per week with** ** type 2 diabetes and oral health issues**			
0	5 (25%)	N/A	-
1–10	15 (75%)		-
11–20	0		-
21–30	0		-
31–40	0		-
>40	0		-
Don’t know	0		-
**Number of patients seen per week with ** **periodontal disease**			
0	N/A	2 (5%)	-
1–10		20 (53%)	-
11–20		6 (16%)	-
21–30		7 (18%)	-
31–40		2 (5%)	-
>40		1 (3%)	-
Don’t know		0	-
**Number of patients seen per week with** ** diabetes or risk factors associated with** ** type 2 diabetes**			
0	N/A	3 (8%)	-
1–10		15 (39%)	-
11–20		12 (32%)	-
21–30		2 (5%)	-
31–40		2 (5%)	-
>40		0	-
Don’t know		4 (11%)	-

Note: n/a = question not asked.

### Survey results

Table 6 shows the survey results. Most GPPs (75%) had no oral health training in their professional education. The majority rarely or never assessed the mouths of patients (70%) and were not confident in identifying oral disease (60%), discussing oral health with their patients (55%) or managing oral health in patients with diabetes (80%).

**Table 6.** Survey results.

Survey items	General practice professionals (n=20) No. (%)	Oral health professionals (n=38) No. (%)
**This was a component of my professional training –**	oral health	connection between oral health and diabetes
Yes	5 (25%)	28 (74%)
No	15 (75%)	2 (5%)
Not sure	0	8 (21%)
**Confidence in identifying –**	signs/symptoms of oral disease in general	risk factors of type 2 diabetes
Very confident/Confident	8 (40%)	25 (66%)
Not confident/Not confident at all	12 (60%)	13 (34%)
**Comfort level in discussing –**	oral health with patients with diabetes	diabetes with patients with periodontal disease
Very comfortable/Comfortable	11 (55%)	31 (82%)
Not comfortable/Not comfortable at all	9 (45%)	9 (18%)
**Confidence in managing patients with diabetes and** ** oral health issues **		
Very confident/Confident	4 (20%)	31 (82%)
Not confident/Not confident at all	16 (80%)	9 (18%)
**I consult with GPs on patients with periodontal** **disease and suspected diabetes**		
Always	N/A	0
Often		2 (5%)
Occasionally		10 (26%)
Rarely		15 (40%)
Never		11 (29%)
**I assess mouths of patients with diabetes**		
Always	1 (5%)	N/A
Often	2 (10%)	
Occasionally	3 (15%)	
Rarely	7 (35%)	
Never	7 (35%)	
**I refer patients with – **	diabetes to an OHP	both periodontal disease and suspected diabetes to a GP
Always	1 (5%)	1 (3%)
Often	4 (20%)	3 (8%)
Occasionally	11 (55%)	12 (32%)
Rarely	3 (15%)	12 (32%)
Never	1 (5%)	10 (26%)
**It should be within my role to undertake simple –**	oral health screening for patients with diabetes	diabetes screening for patients with periodontal disease
Strongly agree/Agree	20 (100%)	37 (97%)
Strongly disagree/Disagree	0	1 (3%)
**Comfort level in conducting simple oral health** **screening**		
Very comfortable/Comfortable	16 (80%)	N/A
Not comfortable/Not comfortable at all	4 (20%)	
**It is feasible in my practice to –**	conduct simple oral health screening for patients with diabetes	use the AUSDRISK screening tool for patients with periodontal disease
Strongly agree/Agree	20 (100%)	35 (92%)
Strongly disagree/Disagree	0	3 (8%)
**Clinical staff in my practice would welcome the** ** introduction of –**	a simple oral health screening tool	the AUSDRISK tool
Strongly agree/Agree	16 (80%)	36 (95%)
Strongly disagree/Disagree	4 (20%)	2 (5%)
**I welcome the opportunity for continuing education/** **training in – **	general oral health	diabetes
Yes	13 (65%)	36 (95%)
No	0	2 (5%)
Not sure	7 (35%)	0
**I am willing to undertake training in – **	oral health screening, advice and referrals for patients with diabetes	diabetes screening, advice and referrals for patients with diabetes or risk factors
Yes	19 (95%)	36 (95%)
No	1 (5%)	2 (5%)
**There is a role for – **	oral health practitioners to screen for diabetes in their dental patients	general practice professionals to screen their patients with diabetes for risk of periodontal disease
Strongly agree/Agree	20 (100%)	37 (97%)
Strongly disagree/Disagree	0	1 (3%)
**Better collaboration between general practice and** ** dental staff would benefit patients with diabetes and** **oral health problems**		
Strongly agree/Agree	19 (95%)	38 (100%)
Strongly disagree/Disagree	1 (5%)	0

In contrast, most OHPs (74%) learnt the relationship between oral health and diabetes in their professional training. The majority were confident in identifying risk factors of type 2 diabetes (66%) and discussing diabetes with their patients (82%) and managing patients with both diabetes and periodontal disease (82%). However, most rarely or never consult GPs (69%). Most GPPs (55%) occasionally referred patients to OHPs while most OHPs rarely or never referred patients to GPPs.

All GPPs agreed that oral health screening was within their role (100%) and most were comfortable to perform simple oral health screening (80%). All thought that oral health screening was feasible in practice (100%) but most thought that it would be welcome by their colleagues (80%). Almost all (95%) welcomed oral health training specifically in diabetes management. All agreed that OHPs should screen patients with periodontitis for diabetes (100%) and almost all thought that better interprofessional collaboration would benefit patients (95%). These results are similar to those from corresponding statements for the OHPs. However, only 65% of GPPs said they would welcome the opportunity for continuing education/training in oral health, compared with 95% of OHPs who said they would welcome continuing education/training in diabetes.

### Interview participants and themes

Five GPPs (four PCNs, three DEs) and 10 OHPs (four Ds, two DHs, two OHTs, one DT and one DA) were further interviewed. Interviews lasted 20 minutes on average. One participant declined to be audio-recorded; none took up the offer to review their transcripts or offer additional feedback.

Data saturation was determined to have been reached. Ten themes were grouped under the three TPB domains and an additional overarching domain to describe participants’ current practice.

**Domain 1: current practice**

***Theme 1: separate diabetes and oral health management***

Most GPP acknowledged that they did not routinely assess the mouth of their patients with diabetes.

“I don’t usually do it routinely unless there is a particular symptom that they complained of or as I am talking to them I can see that they have got an oral health issue” GP2, female, 51–60 years old, worked 11–20 hours per week, 25–30 years’ experience

OHPs on the other hand often discussed the diabetes and oral health link during initial patient examination.

“…if the patient says they have diabetes or has maternal or paternal history of diabetes, I discuss with my patients the risk he and she can have. If he or she has already been diagnosed with gum disease, I inform them about why it’s so important that (their diabetes) should be controlled.” D2, female, 31–40 years old, worked 31–40 hours per week, 6–10 years experience

***Theme 2: poor interprofessional communication or collaboration***

Even where medical and dental services were co-located, they were siloed in practice.

“I have dentists on-site here, but we only really get called when someone is feeling faint. There is little two-way communication.” GP5, female, 51–60 years old, worked 21–30 hours per week, 25–30 years’ experience

***Theme 3: lack of formal referral process***

Most participants tended to refer patients to each other informally.

“I would just ask them if they have seen the dentist. Then they would say yes or no. If they haven’t then I would urge them to go (and) make an appointment with the dentist.” GP2, female, 51–60 years old, worked 11–20 hours per week, 25–30 years’ experience
“So I haven’t referred any patients to a GP directly to get it (diabetes) screened, but I have requested them to see a GP to make sure that their diabetes is under control so I can go ahead with my treatment plan.” D2, female, 31–40 years old, worked 31–40 hours per week, 6–10 years’ experience

GPPs noted that they received little feedback from OHPs following ‘referral’.

“When I refer patients to a physiotherapist or a psychologist, or a cardiologist, I get a letter back. I don’t get anything back from our dental services.” GP1, male, >60 years old, worked 21–30 hours per week, >30 years’ experience

Formal referrals from OHPs to non-GP health professionals were more common.

“I have never referred to a GP for diabetes. We do have diabetes educators… and I would refer for that.” D1, female, 31–40 years old, worked 31–40 hours per week, 11–15 years’ experience

**Domain 2: attitude towards diabetes and oral health management**

***Theme 4: responsibilities and roles***

Many GPPs admitted that oral health was generally overlooked. Many did not think oral health should be their responsibility.

“I don’t think we really know what to do, I think we really leave that to our dental colleagues” GP3, male, 31–40 years old, worked 31–40 hours per week, <5 years’ experience

In contrast, most OHPs thought they should have a role in diabetes screening.

“I think it should be (within our responsibilities). It isn’t though, at the moment.” OHT2, female, <30 years old, worked 31–40 hours per week, <5 years’ experience

However, two dentists expressed apprehension about the ‘unfamiliar territory’ of the Australian Diabetes Risk Assessment (AUSDRISK) tool.

“Another thing is the waist measurement. I don’t know about that. It’s also not really in our place to do so.” D4, female, <30 years old, worked >40 hours per week, <5 years’ experience

GPPs generally agreed that diabetes risk screening is viable in the dental setting.

“…(screening) for diabetes is so simple these days it doesn’t even require a fasting blood test, let alone a glucose tolerance test” GP1, male, >60 years old, worked 21–30 hours per week, >30 years’ experience

Most OHPs also felt GPs and nurses could conduct simple oral health screening and prevention. However, some opposed the idea.

“No, I don’t think (non-dental practitioners should look in patients’ mouth). A doctor can, in a general way. But I don’t think they can make a diagnosis about what the problem is...” DH1, male, 31–40 years old, worked 21–30 hours per week, <5 years’ experience

***Theme 5: further training***

Almost all participants felt further training was needed to improve confidence and competence. However, it needs to be conducive for healthcare professionals to attend.

“But it would need to come out of my paid clinical time and have CPD (continuing professional development) points.” D3, female, 31–40 years old, worked 31–40 hours per week, 6–10 years’ experience

Several participants commented on the value of interdisciplinary education.

“Probably doing things like professional development together, you know, once a year or something like that. That would certainly increase my knowledge… It would also begin to build those working relationships” GP5, female, 51–60 years old, worked 21–30 hours per week, 25–30 years’ experience

***Theme 6: interprofessional collaboration***

Overall, participants recognized the benefits of interprofessional collaboration.

“It shows that we’re creating a united front on the importance of it, and we are taking it seriously and working in collaboration to improve the health of the clients.” OHT1, female, 31–40 years old, worked 21–30 hours per week, 11–15 years’ experience

Many participants however were hesitant about involving time-poor GPs and dentists.

“Yeah, especially between nurses and dental nurses we can be involved. But leave doctors and dentists if they are so busy…” PCN2, female, 41–50 years old, worked 21–30 hours per week, <5 years’ experience

**Domain 3: subjective norms**

***Theme 7: patients’ knowledge and priority of oral health***

Participants thought patients were generally unaware of the relationship between diabetes and oral health.

“Clients are not hugely aware (of the) link of oral health and diabetes, and the bi-directional link…” – OHT1, female, 31–40 years old, worked 21–30 hours per week, 11–15 years’ experience

Some OHPs said that patients did not appreciate the need to discuss diabetes with them…

“There have been a couple of patients who didn’t want to discuss diabetes.” – DH1, male, 31–40 years old, worked 21–30 hours per week, <5 years’ experience

…or prioritized oral health.

“The teeth are the last thing that’s important to them.” – DT1, female, 31–40 years old, worked 21–30 hours per week, 16–20 years’ experience

***Theme 8: perceived resistance from colleagues to change scope of practice***

Many GPPs did not think their fellow colleagues would accept oral health as part of their responsibilities.

“I discussed this with my colleagues just recently, a lot of us believe it’s not really within our scope, and we are not going to venture into an area that we are not that familiar with” DE3, female, 31–40 years old, worked 21–30 hours per week, <5 years’ experience

Many participants contended that the culture of siloes was a barrier.

“I think it’s just the way the (health) profession has been for so long. Each person just does their own thing, and there’s no collaboration.” DT1, female, 31–40 years old, worked 21–30 hours per week, 16–20 years’ experience

**Domain 4: perceived behavioral control**

***Theme 9: lack of opportunity for training***

Participants highlighted a lack of opportunities for further training.

“I have had absolutely no training on dental health apart from growing up in a family where we were trained to brush our teeth” – GP5, female, 51–60 years old, worked 21–30 hours per week, 25–30 years’ experience

Many participants perceived that their availability for training was in fact not within their control.

“It depends on my manager… how much she can provide us with the training hours.” PCN2, female, 41–50 years old, worked 21–30 hours per week, <5 years’ experience

***Theme 10: systemic barriers***

Time constraint was a barrier for almost all participants.

“Time is a huge issue. I have mostly half-an-hour appointments, which is a very limited scope for me because I have other things to do as well… To include everything in that half an hour would be very tough and a bit of a problem.” DH1, male, 31–40 years old, worked 21–30 hours per week, <5 years’ experience

This excuse, however, was quashed by other participants.

“It doesn’t take that long to do and we can do it. I have been listening to people say that “We don’t have time to do it”, but I think that we can just make time. It’s an important thing to do.” OHT1, female, 31–40 years old, worked 21–30 hours per week, 11–15 years’ experience

Some participants thought that the lack of software uniformity and integration of information technology between professions hampered collaboration.

“Dental files are dental files and medical files are medical files. … the only person you’re relying on is what the patient relays back to you, and sometimes they don’t even know what’s being told to them except use this medication, get your dental check-up on this day.” – DT1, female, 31–40 years old, worked 21–30 hours per week, 16–20 years’ experience

High dental costs and long public dental waiting list were the most common reasons that GPs, PCNs and DEs gave for their reluctance to refer patients to OHPs.

“Another barrier is cost… (Patients) are so used to bulk-billing and they thought that if medical bulk bills, why not dental as well.” PCN2, female, 41–50 years old, worked 21–30 hours per week, <5 years’ experience
“…even the minor delay of even a week or two is sufficient for the patient to scurry away and say I’ll do it another time, and then the opportunity is lost.” GP1, male, >60 years old, worked 21–30 hours per week, >30 years’ experience

## Discussion

Our research aims align with the National Oral Health Plan’s recommendations for greater collaboration of OHPs with the broader health workforce ^23^. Our findings contribute to a growing evidence base for interprofessional collaboration between medical and oral health professionals and will help support the RACGP guidelines on diabetes management and IDF guidelines on interprofessional collaboration ^9, 10^. This corresponds with the recommendations from a UK study to develop initiatives and policies to promote and embed oral health management as part of diabetes care ^24^.

The TPB model provided the framework to explain the key factors influencing healthcare professionals’ consideration of interprofessional care of diabetes and periodontitis ^21^. Several attitudinal beliefs and societal normative influences strongly impact their collaborative behavioral patterns. Our results are similar to those from studies that have found many non-oral healthcare professionals do not manage the oral health of patients with diabetes ^25, 26^.

Like our study, a German study also reported a lack of collaboration from OHPs which was likely a result of the informal nature of ‘verbal referrals’ usually directed at OHPs ^27^. Other research shows that OHPs supported diabetes screening becoming part of oral health professionals’ standard care but the convoluted referral system dissuaded them from providing formal referrals ^8^. It is important that a simple and structured referral system, like the one between medical specialists, be developed between medical/nursing practitioners and OHPs to promote effective interprofessional collaboration.

Currently, Australian medical and dental practices use completely different information systems that are not integrated. This compounds service fragmentation and suboptimal clinical outcomes. Appropriate policies are required to incorporate information sharing in health systems to support interprofessional collaborative relationships ^28^.

It was not surprising that time constraint was a barrier particularly for GPs and dentists. They may be more suited to be involved after the initial primary prevention strategies. The barrier of the healthcare profession ‘silo’ culture is well-known and is also reflected in Marshall and Spencer’s paper which cites a “separateness” between Australian medical and dental practices ^29^. However, improved management of periodontitis would potentially improve blood glucose control, which would in turn further improve periodontal health resulting in longer-term fewer visits to GP and dental clinics and ultimately save time and resources.

Further training in diabetes and oral health management would increase healthcare professionals’ knowledge and confidence ^30^. Ward *et al.* found that nurses who were confident with their oral health education were more likely to screen patients with diabetes for periodontitis ^25^. The importance of interdisciplinary training is consistent with Lamster and Eaves’ push for greater interprofessional collaboration and emphasis on respecting all health disciplines, increasing the understanding of each profession’s role, providing more effective communication and maximizing safety, efficiency and effectiveness ^11^. Currently there are minimal interprofessional training opportunities. Development of future training should have an interprofessional focus, be as conducive as possible and be accredited for CPD.

### Strengths and limitations

Our mixed-methods approach allowed an in-depth exploration of participants’ views. Although the sample size was small, the wide range of healthcare professionals provided broad perspectives. Unequal representation from different professional groups may impede the generalizability of the findings even though data saturation was reached. Our focus on CHSs with co-located general practice and dental services may have limited the extrapolation of our findings to other settings.

## Conclusion

Primary healthcare professionals generally recognized the importance and have strong intentions to engage in interprofessional diabetes and oral health management. Accredited interprofessional training should bridge the divide between medicine and dentistry. Formal referral processes are necessary to improve interprofessional feedback and communication. Health policies and advocacies need to target dental costs and public dental waiting lists to motivate referrals. An effective and feasible interprofessional collaborative diabetes and oral health care model would contribute to improved patient outcomes. Future studies should include the views of patients, policy makers and other stakeholders.

## Data availability

### Underlying data

Deidentified data of this research will only be provided on request. Reviewers or other researchers intending to reproduce the study may make this request by emailing the corresponding author. This conditional withholding of data is necessary to protect the privacy and confidentiality of the participants who were sourced from a small number of community health services and the final sample size was small.

### Reporting guidelines

Figshare: COREQ checklist for ‘Interprofessional diabetes and oral health management: what do primary healthcare professionals think?’ https://doi.org/10.26188/14454372.

Data are available under the terms of the Creative Commons Attribution NoDerivatives 4.0 International license (CC-BY-ND 4.0).
