# Supplementary material for: Interprofessional diabetes and oral health management: what do primary healthcare professionals think?
Source: F1000Res. 2021 May 4;10:339. [Version 1] doi: 10.12688/f1000research.52297.1 (PMC8647041; doi:10.12688/f1000research.52297.1)
Supplement: Peer review history

# Peer review: Reviewer response for version 1

**DOI:** 10.5256/f1000research.55554.r98065

**Published:** 2021-12-02

**Authors:** Shahida Mohd-Said

**Affiliations:** 1 Centre for Restorative Dentistry, Faculty of Dentistry, Universiti Kebangsaan Malaysia, Kuala Lumpur, Malaysia

The paper describes the oral health care professionals' qualitative input on their personal experiences, knowledge, attitude, and interprofessional collaboration when managing diabetes in Melbourne, Australia. The aim of the study is relevant and critical, and generally, it was conducted using a comprehensive, relevant, good, and clear methodology as well described in the paper.  Perhaps the inclusion of more references within 2018-2021 would benefit this paper since more closely relevant qualitative studies have been published recently involving health professionals and concepts in interprofessional or shared care for patients with diabetes.

 Revision to the main conclusion would be beneficial to reflect the study findings more accurately rather than generalising the statement to all primary healthcare professionals.

Is the work clearly and accurately presented and does it cite the current literature?

Partly

If applicable, is the statistical analysis and its interpretation appropriate?

Not applicable

Are all the source data underlying the results available to ensure full reproducibility?

Yes

Is the study design appropriate and is the work technically sound?

Yes

Are the conclusions drawn adequately supported by the results?

Yes

Are sufficient details of methods and analysis provided to allow replication by others?

Yes

Reviewer Expertise:

Oral Health Management Innovation Research: Teledentistry and Integrated Care Management of Periodontitis Patients

I confirm that I have read this submission and believe that I have an appropriate level of expertise to confirm that it is of an acceptable scientific standard.

# Peer review: Reviewer response for version 1

**DOI:** 10.5256/f1000research.55554.r84496

**Published:** 2021-05-18

**Authors:** Judith Haber

**Affiliations:** 1 New York University Rory Meyers College of Nursing, New York, NY, USA

This is a well-designed mixed methods study that is based on a current review of the literature. The focus of the study highlights a significant global population health problem, diabetes and periodontal disease and seeks to identify the potential barriers and facilitators of effective interprofessional care for this population. The surveys and interview guides are well constructed, the data analysis appropriate, and the findings are reported in a very understandable way that elucidates their potential applicability in education and practice.

 I would accept this well written manuscript and am confident that it will be an important contribution to the literature both in Australia and the United States.

Is the work clearly and accurately presented and does it cite the current literature?

Yes

If applicable, is the statistical analysis and its interpretation appropriate?

Yes

Are all the source data underlying the results available to ensure full reproducibility?

Yes

Is the study design appropriate and is the work technically sound?

Yes

Are the conclusions drawn adequately supported by the results?

Yes

Are sufficient details of methods and analysis provided to allow replication by others?

Yes

Reviewer Expertise:

Relationship of Oral Health to Overall Health

I confirm that I have read this submission and believe that I have an appropriate level of expertise to confirm that it is of an acceptable scientific standard.

# Response

**Published:** 2021-05-18

**Authors:** Phyllis Lau

**Affiliations:** University of Melbourne, Australia

We thank the reviewer for her very positive and encouraging comments. Much appreciated!
